# Blackmailing: the keystone in the human mating system

**DOI:** 10.1186/1471-2148-11-345

**Published:** 2011-11-29

**Authors:** Milind G Watve, Anuja Damle, Bratati Ganguly, Anagha Kale, Neelesh Dahanukar

**Affiliations:** 1 Indian Institute of Science Education and Research, Sai Trinity Building, Pashan, Pune 411021, India; 2 Anujeeva Biosciences Pvt. Ltd., 10, Pranav Society, 1000/6-c Navi Peth, Pune 411030, India; 3 Department of Chemistry and Molecular Biology, North Dakota State University, 1231 Albrecht Avenue, PO Box-5516, Fargo, ND 58105-5516, USA

## Abstract

**Background:**

The human mating system is characterized by bi-parental care and faithful monogamy is highly valued in most cultures. Marriage has evolved as a social institution and punishment for extra pair mating (EPM) or adultery is common. However, similar to other species with bi-parental care, both males and females frequently indulge in EPM in secrecy since it confers certain gender specific genetic benefits. Stability of faithful monogamy is therefore a conundrum. We model human mating system using game theory framework to study the effects of factors that can stabilize or destabilize faithful committed monogamy.

**Results:**

Although mate guarding can partly protect the genetic interests, we show that it does not ensure monogamy. Social policing enabled by gossiping is another line of defense against adultery unique to humans. However, social policing has a small but positive cost to an individual and therefore is prone to free riding. We suggest that since exposure of adultery can invite severe punishment, the policing individuals can blackmail opportunistically whenever the circumstances permit. If the maximum probabilistic benefit of blackmailing is greater than the cost of policing, policing becomes a non-altruistic act and stabilizes in the society. We show that this dynamics leads to the coexistence of different strategies in oscillations, with obligate monogamy maintained at a high level. Deletion of blackmailing benefit from the model leads to the complete disappearance of obligate monogamy.

**Conclusions:**

Obligate monogamy can be maintained in the population in spite of the advantages of EPM. Blackmailing, which makes policing a non-altruistic act, is crucial for the maintenance of faithful monogamy. Although biparental care, EPM, mate guarding and punishment are shared by many species, gossiping and blackmailing make the human mating system unique.

## Background

Cooperation is commonly accompanied by cheating or defection in a number of naturally occurring social systems [1-4]. In Prisoner's Dilemma (PD), the popular model of cooperative behaviour, cooperation is not stable and defection is the only stable strategy for any player [5]. However, in iterated PD games strategies involving reciprocity or reputation can help stabilize cooperation [5,6]. If the same individuals play the game again, defection can be retaliated [5]. Furthermore, cooperators can build a reputation and derive long term gains from it [6]. Cooperation can also evolve if co-operators punish those who defect [7-9]. However, a potential problem in punishment is that there is a cost in executing punishment, which makes punishment an altruistic act. Since the benefits derived by punishing cheaters are shared, there arises a possibility of second order free riders that cooperate but do not contribute to punishment. The second order free riders can destabilize punishment and cooperation in turn. A number of conditional solutions to this problem have been suggested [8-10].

One of the basic assumptions behind all suggested solutions to the problem of stability of cooperation is that the strategies of players and the resulting payoffs are open at the end of every interaction. Retaliation, punishment or reputation is possible only if players have access to the history of other players and would fail to work for games in which the strategies and payoffs remain hidden. In a number of real life games it is possible that the players have a choice of secrecy or deception. Evolution is likely to take a different path when the strategies as well as payoffs remain hidden from the players most of the time.

The human mating system is an ideal and obvious example of hidden strategy games. Since paternity is uncertain the actual reproductive success of a male remains hidden. For females although some of the components of benefits, for example resource partitioning and the number of offspring borne by them is obvious, the genetic contributor to the offspring and therefore the benefit of "good genes" remains hidden. It is possible to apply game theoretic models human mating system considering faithful long term bi-parental care as cooperation and extra-pair mating (EPM) or cuckoldry as defection [11]. In a number of species where biparental care is necessary, EPM or cuckoldry is known to be common [12-21]. Human marriage system is more complex than other species with bi-parental care in that marriage is not only a game of cooperative partnership but it is a social institution. Although societal rules differ regarding the allowed number of spouses at a time [22], long term investment and faithful commitment by both genders in childcare is central to the system. Further, faithful monogamy is highly valued in most cultures. There are likely to be multiple reasons for the evolution of a committed marriage system, the main reason being the prolonged dependence of the human child. Biparental care and division of labor is necessitated by the extreme human newborn altriciality, demand of multiple simultaneous dependents and longer period for maturity [23,24]. As a general rule, sexual dimorphism in size is shaped by the mating system, with most promiscuous species showing maximum dimorphism and those with monogamy and biparental care showing little dimorphism. In human evolution, size dimorphism is low through most of the *Homo *and pre-homo lineage indicating that mating system could be stable over time. It is reasonable to assume therefore that monogamy is evolutionarily ancient in hominids [25]. Although biparental care necessitates cooperation, it may not be sufficient to maintain faithful monogamy as occult polygamy can confer added genetic benefits to both the genders. Thus, similar to other species with biparental care, EPM or cuckoldry is known to occur at varying frequencies in the human society as well [12,18,20,21,26,27]. In cuckoldry, the polygamous individuals get an additional genetic advantage, but their mates have to bear a loss. Males can increase their number of offspring by indulging in EPM. The benefits are not in terms of number of offspring for females. The advantage of EPM for females can be in terms of additional resource acquisition, sperm competition or getting dual benefit of good parenting from one male and good genes from another [20,28-31]. Males suffer a direct genetic loss by their partner's EPM since their paternity is threatened [26]. The loss to females is less obvious since their maternity is never threatened. However, they may suffer in other ways like loss of the man's time, attention, energy, parenting investment, and commitment [19,21].

The human mating system as a hidden strategy game differs from the games of incomplete information [32] in that, not only the strategy and payoff of the other player is hidden but the payoff of the focal player also remains hidden. Since there are few opportunities to learn from payoffs, learning is less likely to shape the behaviour but natural selection would certainly continue to act at the genetic level. Therefore human sexual behaviour is more likely to be dominated by instincts rather than learning.

Two types of measures against cuckoldry are seen in the human society, namely mate guarding [19,20], a trait shared by many species [33-36], and societal punishment if cuckoldry gets exposed [11] which appears to be uniquely human. Although the probability of getting exposed is small its consequences are known to be severe in most human societies and exposed adulterous individuals generally receive punishment in some or the other form. Altruistic punishment or strong reciprocity has been used in the models of evolution of cooperation [7,8,37]. However, altruistic punishment suffers from the problem of second order free riders [37,38]. In the human mating system there can be a non-altruistic punishment in the form of social sanctions. If an adulterous individual is deserted by its partner who makes the reason public, the probability of pairing again could be very small for the deserted individual, a form of ostracism or community boycott [39,40]. While for the deserting partner, if the probability and the net benefit of pairing again is higher than the net benefit from continuing partnership with a known defector, deserting would be a non-altruistic way of punishing. Avoiding pairing with a known defector is also a non-altruistic act. Therefore, punishment for cuckoldry by the partner as well as by the society need not have an altruistic element in it at least in its minimal form. More complex punishment systems appear to have evolved in complex societies but the basic form of minimal punishment in terms of social sanctions can be considered non-altruistic.

However, since individuals have the choice of secrecy, if the probability of getting exposed remains small, punishment may fail to curb EPM. The stability of monogamy would depend upon mechanisms to increase the probability of exposure. In humans, due to evolution of language, gossip is possible through which one can gain information about the behaviour of a sexual partner in one's absence. In fact the need to gossip has been argued to be one of the major selective forces for the evolution of language [41]. This is an indirectly reciprocating, apparently altruistic social act that forms a component of 'social policing'. Social policing consists of making observations about others' EPM and its indicators on the one hand and gossiping about it on the other. The cost involved in social policing can be substantially small as compared to individual mate guarding. This is because in individual mate guarding one has to compromise on foraging and other activities for keeping a watch on the mate, while social policing is more of an incidental act which an individual does during its regular activities resulting in lower costs. However, as long as there is a non-zero cost of social policing some second order free riders can take advantage of the system. Individuals who do not contribute to social policing may still gain from it by getting information about their sexual partner. Such free riders can destabilize social policing.

We suggest here that opportunistic blackmailing can give a solution to the problem. Since the strategies are hidden, and exposure can lead to punishment, blackmailing is possible. When defection is exposed to only one or a few individuals the defector may give some form of direct benefits to the blackmailer and avoid social punishment. Since blackmailing necessarily depends upon differential secrecy, it is restricted to hidden strategy games. We use the definition of blackmailing given by Blocks [42], which is the demand for money or other valuable consideration, coupled with an offer to refrain from exposing a secret which is embarrassing to the blackmailee. The success of blackmailing is highly conditional, but whenever the conditions favour blackmailing, it can give direct returns on investment in policing. Policing without blackmailing is an altruistic act and will be selected against in the presence of blackmailers. For a policing individual, the opportunities to blackmail increase with increasing investment in social policing, in particular the observation component of policing. Since non-policing individuals are less likely to get a blackmailing opportunity, free riders are unlikely to thrive. All individuals engaged in EPM, on the other hand, have to bear a probabilistic penalty as a result of social punishments or blackmailing. Thus, opportunistic blackmailing makes social policing a credible threat. We show here that inclusion of blackmailing in a hidden strategy game leads to stable or oscillatory coexistence of different strategies, where cooperation is maintained at high proportions. On the other hand omission of blackmailing from the model leads to collapse of the policing and punishment systems and occult polygamy is the only ESS possible. We have further shown that constraints on blackmailing, imposed by the social norms and judiciary systems, do not affect the results of the model.

## Methods

### Model

Our model assumes a "marriage system" in which every individual player has to engage in a cooperative and committed parenting act. We use the word monogamy here to indicate faithful and committed partnership. The central concept would be applicable also to societies where more than one spouses are allowed as long as there is faithful committed parenting but we assume one spouse partnership in the model to avoid complexity. The baseline model assumes that all individuals are socially monogamous, but they can become genetically polygamous by engaging in EPM. Players have a choice to desert and probabilistically pair again exerting a choice to deny pairing with individuals with a bad reputation. Individual players in the model can have alternative behavioral options on four different lines. Individuals can be (i) genetically monogamous (M^+^) i.e. co-operators or polygamous (M^-^) i.e. defectors (ii) guarding (G^+^) or non-guarding (G^-^) (iii) policing (P^+^) or non-policing (P^-^) and (iv) blackmailers or non-blackmailers. Where, policing trait includes both observation and gossiping. Combinations of the above traits give 16 different behavioral options. However, policing without blackmailing is at an all time disadvantage as compared to policing blackmailers. This is because policing is an altruistic act and therefore blackmailing police always invade altruistic police as long as there is a non-zero positive benefit of blackmailing. On the other hand since blackmailing can be done only if the individuals have selective information, and policing is the best way to gain information, non-policing blackmailers are unlikely to survive. This results into an obligate association between policing and blackmailing and leaves only 8 pure strategies in the game *viz*. M^+^G^+^P^+^, M^+^G^-^P^+^, M^+^G^-^P^-^, M^+^G^+^P^-^, M^-^G^+^P^+^, M^-^G^-^P^+^, M^-^G^-^P^- ^and M^-^G^+^P^- ^where P^+ ^are policing individuals who are also involved in opportunistic blackmailing while P^- ^are non policing non blackmailers (Table 1).

**Table 1:** List of parameters with description and symbol.

Description	Symbol
Genetically monogamous individual	*M^+^*
Genetically polygamous individual	*M^-^*
Player engaged in individual mate guarding	*G^+^*
Player who does not do individual mate guarding	*G^-^*
Player involved in policing and opportunistic blackmailing	*P^+^*
Player not involved in policing and blackmailing	*P^-^*
Probability that a player is genetically monogamous	*p*
Probability that a player guards its mate	*q*
Probability that a player is involved in social policing and blackmailing	*r*
Additional payoff due to EPM	*z*
Loss due to the partner's EPM	*l*
Cost of guarding one's own mate	*c_G_*
Reduction in *l *because of mate guarding	*s*
Cost of social policing	*c_P_*
Benefits from cooperative social policing	*α*
Reduction in cuckoldry due to social policing	*β*
Punishment penalty to punished individual	*y*
Benefits of opportunistic blackmailing	*a*

We assume that the fitness of both the partners in a strictly monogamous, non-guarding, non-policing pair is unity. This is taken as the baseline fitness for the model. All polygamous individuals have an additional maximum possible advantage *z *as a result of extra pair mating. In practice *z *is limited by the opportunities for EPM which are proportional to the availability of polygamous sexual partners ready for EPM. The partners of individuals indulging in EPM have to bear a loss *l*. The loss *l *could consist of several components including direct genetic loss due to cuckoldry or loss in parenting resources coming from the partner. If the population is perceived as obligately paired individuals, EPM cannot be a one sided act. The probability of getting an EPM partner will depend upon the proportion of opportunistically polygamous individuals. Therefore the realized benefit (*z*) and loss (*l*) would be proportional to polygamous individuals. The loss from cuckoldry for any individual depends on the proportion of adulterous individuals in a dual way. The probability of one's partner being polygamous as well as the probability that he/she gets an EPM partner both depend on it. However, if we depart from the ideal population where each individual is in a partnership, which can be true for populations with skewed sex ratios, virgin individuals or immigrants, there could be some benefit of EPM even in the absence of other polygamous individuals.

Individuals actively guarding their mates incur a cost of guarding *c_G_*, and as a result of guarding could reduce their loss *l *by a fraction *s*. It is obvious that guarding would exist only if *l.s *>*c_G_*. We assume that the cost of policing *c_P _*<*c_G _*since for individual mate guarding one has to give extra efforts for watching the mate, while social policing is incidental to the regular activities. The benefit of social policing *α *is availed by all individuals alike and is assumed to be directly proportional to the fraction of policing individuals in the society. Thus, the loss *l *is reduced in proportion to fraction of policing individuals in the society. Reciprocally, for polygamous individuals their benefit *z *is reduced by a fraction *s *when the partner is guarding. As a result of social policing, there is a reduction *β *in the success of cuckoldry that is directly proportional to the fraction of policing individuals in the society. Policing has a dual function, as on the one hand it prevents EPM; on the other it exposes adultery. The adulterous individuals are assumed to get a punishment *y *if exposed, the probability of which is in direct proportion to the fraction of policing individuals in the society. The term *y *includes the probability of being exposed and receiving punishment, the direct loss in reproduction owing to deserting by the partner, a bad reputation resulting into reduced probability of pairing again or alternatively the probability of being blackmailed. For a policing individual the opportunities to blackmail are assumed to increase linearly with the number of polygamous individuals in the population. Hence, blackmailing could give direct returns to the policing individuals proportional to the fraction of all polygamous individuals, the proportionality constant being *a*. We keep *a < y *throughout the model.

If we assume that the entire population consists of socially monogamous pairs with no immigration or emigration, we can maintain consistency in the payoff structure as suggested by Houston and McNamara [43,44], by keeping *z *= *l *and *α = β*. However, we have chosen to use different symbols for benefits (*z *and *α*) and losses (*l *and *β*) to maintain the flexibility in terms of modelling so as to study the effect of non consistent payoffs. Non-consistent pay-offs are possible if the population has virgin individuals, sex ratio is skewed or there is immigration or emigration.

A player could have a mating strategy with varying degrees of monogamy, guarding and policing traits. In other words players can adapt strategies in continuous strategic space. Each player is characterized by a strategy consisting of three probabilities (*p*, *q*, *r*) ∈ [0, 1]^3^, *p *being the probability that a person behaved monogamously, *q *the probability that the person guarded his/her mate and *r *the probability that the person was involved in social policing and opportunistic blackmailing. Thus, the payoff of a person using a strategy (*p_1_*, *q_1_*, *r_1_*) in a population using the strategy (*p_0_*, *q_0_*, *r_0_*) would be

(1) $$\begin{array}{c} E\left[\left(p_{1},q_{1},r_{1}\right)|\left(p_{0},q_{0},r_{0}\right)\right]=p_{1}\left(1\right)+\left(1-p_{1}\right)\left(1+z\left(1-p_{0}\right)\right)+{\left(1-p_{0}\right)}^{2}\left(-l\right)+q_{1}\left(-c_{G}\right)+q_{1}{\left(1-p_{0}\right)}^{2}\left(l.s\right)\\ \;\;\;\;\;\;\;\;\;\;\;\;+q_{0}\left(1-p_{1}\right)\left(-z\left(1-p_{0}\right).s\right)+r_{1}\left(-c_{P}\right)+r_{0}{\left(1-p_{0}\right)}^{2}\left(\alpha .l\right)\\ \;\;\;\;\;\;\;\;\;\;\;\;+r_{0}\left(1-p_{1}\right)\left(-\beta .z\left(1-p_{0}\right)\right)+r_{0}\left(1-p_{1}\right)\left(1-p_{0}\right)\left(-y\right)+r_{1}{\left(1-p_{0}\right)}^{2}\left(a\right) \end{array}$$

All possible combinations of the (*p*, *q*, *r*) strategy can be represented by a unit cube where the corners are the eight pure strategies we discussed earlier while any other combination of (*p*, *q*, *r*) strategy that lie on the edges or in the interior of the cube is a mixed strategy of the game.

We will study the model under two scenarios. First, assuming that the gains and losses do not vary across gender we will study how monogamous traits will invade the population using invasion dynamics and adaptive dynamics. If sexes do not differ in payoffs and sex ratio is unity, the two sexes need not be modeled separately. Second, by considering that the gains and losses differ for genders we will study the gender difference model using replicator dynamics where the dynamics of the two sexes needs to be modeled separately.

### Invasion dynamics

Invasion of population using one pure strategy by a mutant using another pure strategy was determined using the logic of evolutionary stable strategies (ESS) proposed by Maynard Smith and Price [45]. A rare mutant strategy *j *will invade a population of players using strategy *i *if the payoff strategy *j *in the population of *i*, i.e. *E*[*j*|*i*], followed the inequity *E*[*j*|*i*] >*E*[*i*|*i*].

### Adaptive dynamics

We used adaptive dynamics [46] formulated as a differential equation in the space of all strategies [47] to understand the dynamics of all mixed strategies in the game. Let there be *n *different behavioral options for each player and each strategy can be described by some continuous parameters *p*_1_,...,*p_n_*, where *p_i _*∈ [0, 1]*^n^*, and that the entire population plays strategy *S = S*(*p*_1_,..., *p_n _*). The expected payoff received by a mutant *S' = S'*(*p'*_1_,...,*p'_n_*) is given by *E*[*S'*|*S*]. The adaptive dynamics are given by the full system of differential equations

(2) $${\dot{p}}_{i}={\left(\frac{\partial E\left[S^{\prime }|S\right]}{\partial p_{i}^{\prime }}\right|}_{S^{\prime }\to S}$$

Where *i *= 1,2,...*n*. The system of differential equations for the human mating game using payoff structure in equation (1) can be given as follows,

(3) $$\dot{p}=\left(1-p\right)\left[ry-z\left(1-s.q-\beta .r\right)\right]$$

(4) $$\dot{q}=-c_{G}+l.s{\left(1-p\right)}^{2}$$

(5) $$\dot{r}=-c_{P}+a{\left(1-p\right)}^{2}$$

These differential equations can be used in adaptive dynamics framework to understand the evolution of triplet strategies.

### Replicator dynamics

Because of the limitations in representing the results of the adaptive dynamics for higher dimensional system, we could not use adaptive dynamics for gender difference model as it meant that we had to deal with total 16 strategies, 8 each for males and females. We therefore used replicator dynamics to study gender difference model. Using payoff structure given in equation (1) the linear fitness functions $E_{i}=\sum_{j}f_{j}E\left[i|j\right]$ was derived where *f_j _*is the frequency of players using strategy *j*. To simulate how frequencies of the players using the given strategy changed, we used replicator equation ${\dot{f}}_{i}=f_{i}\left(E_{i}-\bar{E}\right)$ [ref. [46]]. The replicator equation describes deterministic but frequency-dependent selection dynamics. The fitness, *E_i_*, of type *i *is a function of the frequencies of all strategies (phenotypes) $\overset{⇀}{f}=\left(f_{1},f_{2},......,f_{n}\right)$. The average payoff of the population is given by $\bar{E}=\sum_{i}f_{i}E_{i}$. Two genders, males and females, were assumed and there was random pairing between individuals of opposite genders. We assumed different costs (*l*) and benefits (*z*) of EPM for the two genders. Since the fitness values can be considered as gender specific relative fitness scores, it is not necessary for the gender difference model that the average fitness of males and females remains the same.

## Results

### Dynamics between the pure strategies

**Figure 1:**
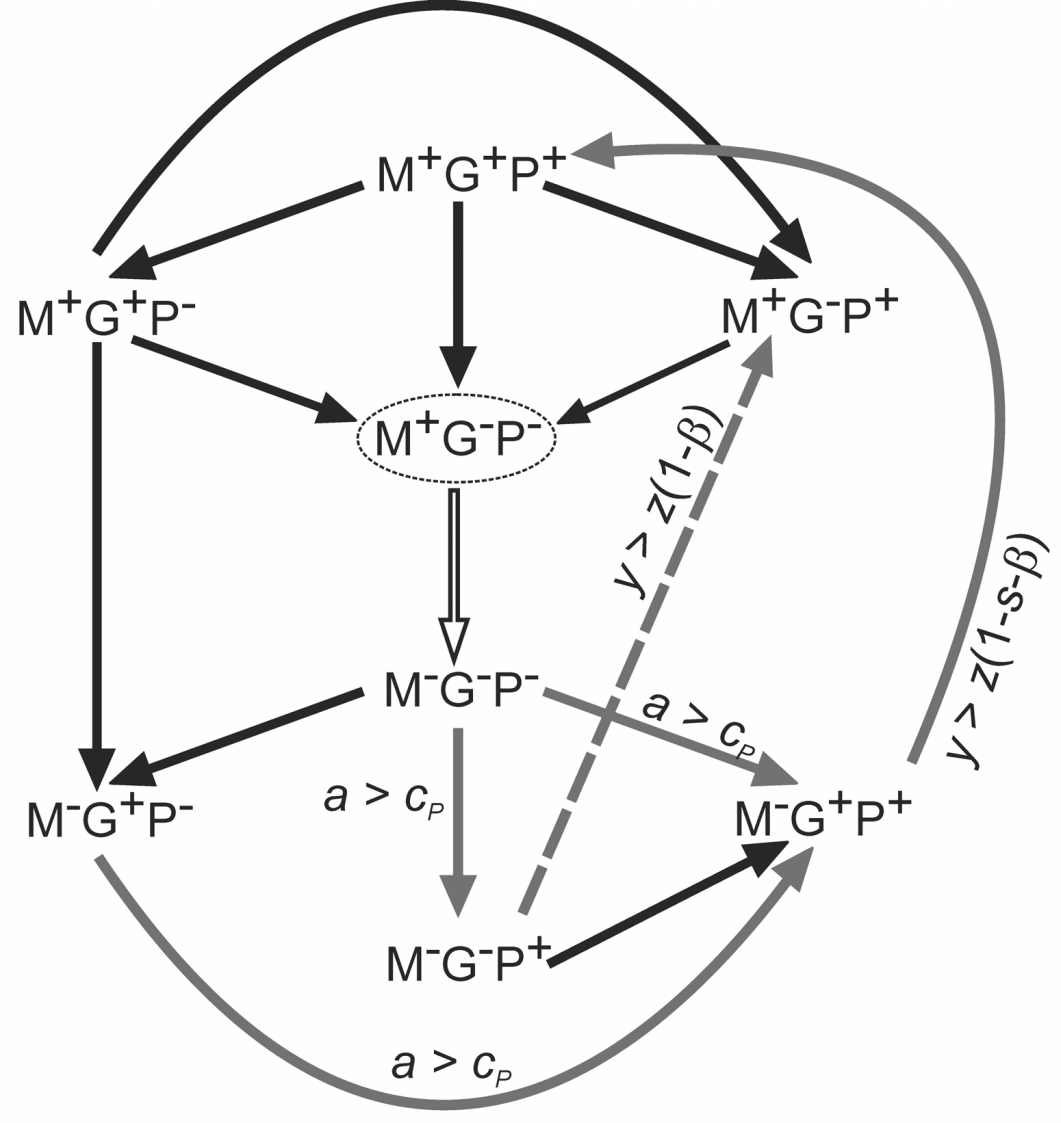
**Invasion dynamics of 8 pure strategies**. Black arrows indicate that invasion is possible through basic assumptions of the model. Grey arrows indicate that invasion is possible only under certain conditions. Arrows point at the invading strategy. The circled strategy is an unstable equilibrium. The hollow arrow indicates that the payoff of a lone invader is the same as the population, however even with slight drift invasion is possible.

Invasion dynamics between 8 pure strategies (Figure 1) shows that each strategy can invade at least one other strategy and gets invaded by at least one other strategy with the exception of M^+^G^-^P^-^. M^+^G^-^P^- ^is a local or unstable equilibrium since in this pure population a polygamous invader will fail to find an EPM partner and therefore can get no advantage. However, since M^-^G^-^P^- ^has the same fitness in a M^+^G^-^P^- ^population, invader may grow by drift alone. With adequate proportion of the invader, the benefit of polygamy would allow invasion by M^-^G^-^P^-^. In the presence of polygamy both guarding and policing traits have an advantage and start increasing. Mate guarding increases because guarding one's own mate reduces the chances of defection by the partner. Policing trait increases because it gives an opportunistic benefit of blackmailing. If the benefit of blackmailing is more than the cost of policing (*a *>*c_P_*) then all polygamous traits are invaded by polygamous guarding policing trait M^-^G^+^P^+^. If the punishment is sufficiently large (*y *>*z*(1-*s*-*β*)), then M^+^G^+^P^+ ^can invade the population. However an obligate monogamous guarding and policing strategy M^+^G^+^P^+ ^is vulnerable to invasion by strategies which do not have policing or guarding traits. This is because, in the absence of polygamy costly guarding and policing is selected against. As a result, in the absence of polygamy, policing and guarding traits disappear increasing the proportion of M^+^G^-^P^- ^and the cycle continues. As a result when the condition *y *>*z*(1-*s*-*β*) is satisfied all the eight strategies coexist in oscillations. But if *y *is still larger such that *y *>*z*(1-*β*) then only four strategies namely, M^+^G^-^P^+^, M^+^G^-^P^-^, M^-^G^-^P^+^, and M^-^G^-^P^- ^oscillate. The last result suggests that monogamy can be maintained even in the absence of mate guarding but in the presence of social policing and opportunistic blackmailing.

### Dynamics in mixed strategy space

**Figure 2:**
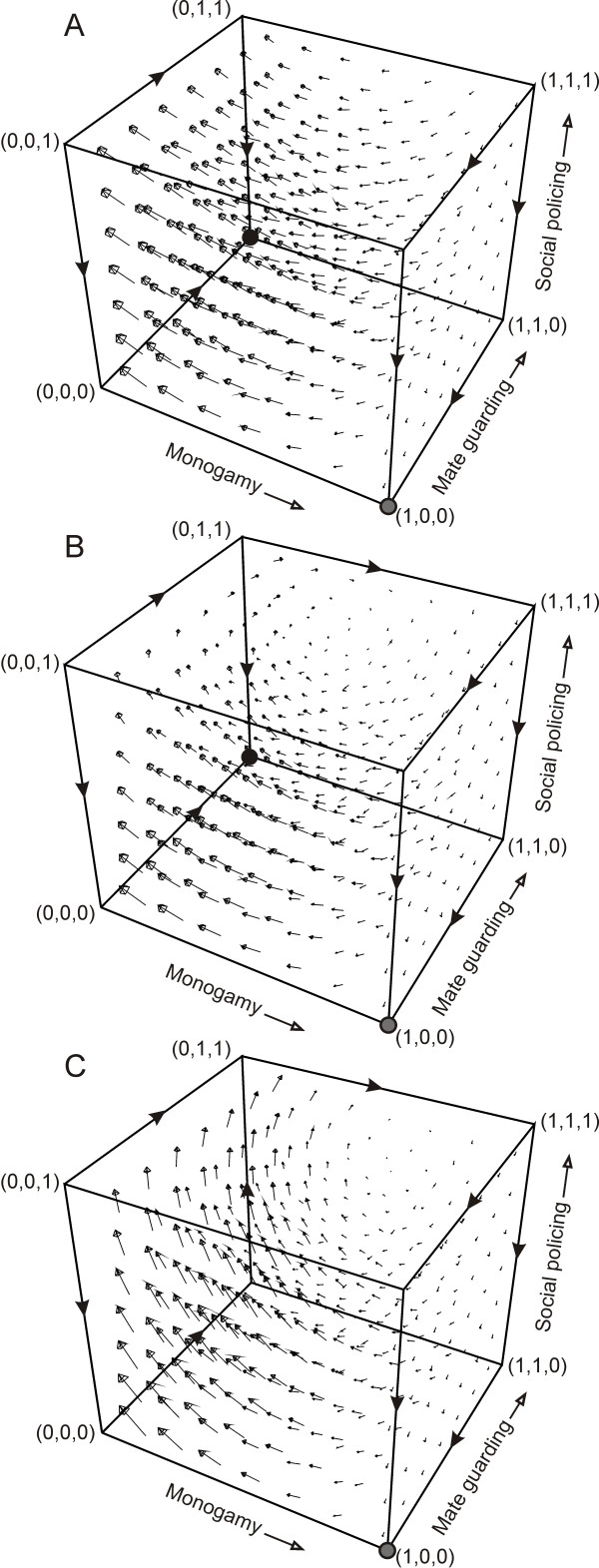
**Vector field plots of differential equations (2), (3) and (4)**. (A) in the absence of punishment and blackmailing (*y *= 0, *a *= 0), (B) in the presence of punishment but no blackmailing (*y *> 0, *a *= 0) and (C) in the presence of punishment and blackmailing (*y *> 0, *a *> 0). Arrows indicate the direction of change in strategy. Grey circle indicates unstable equilibrium and black circle indicates stable equilibrium. (A) and (B) end up at the stable equilibrium (0,1,0) whereas (C) leads to cyclic oscillations in strategies with no ESS. Other parameter values are *l *= z = 0.2, *α *= *β *= 0.2, *c_P _*= 0.01, *c_G _*= 0.02, *y *= 0.1, *s *= 0.4 and *a *= 0.08.

We will now explore the dynamics of the entire strategic space. Simple analytical solutions reveal that in the absence of punishment and blackmailing (*y *= 0, *a *= 0), the unique equilibrium of the game consists of the strategy (0, 1, 0) (Figure 2A). This could be depicted from equations (3), (4) and (5). At *a *= 0, *r *approached zero for any *c_P _*> 0, so also, as *r*.β.*z *→ 0, *p *also approached zero since *q*.*z*.*s *<*z *for every 1 ≥ *q *≥ 0 and *s *< 1. However, as we assumed that *l.s *>*c_G_*, *q *increases in the population and gets fixed. Similarly, in the presence of punishment but absence of blackmailing (*y *> 0, *a *= 0) the equilibrium is fixed at (0, 1, 0) where both *r *and *p *approach zero for any *c_P _*> 0 and 1 ≥ *q *≥ 0, *s *< 1, while *q *increases in the population and gets fixed at *q *= 1 (Figure 2B). The only difference in the later case as compared to the former case is that monogamy invades polygamous population if the initial population of policing individuals is very high. However, this invasion is temporary since *r *always decreases and in the absence of policing monogamy fails to establish in the population. Thus, the only stable equilibrium in both the cases is (*p*, *q*, *r*) = (0, 1, 0).

**Figure 3:**
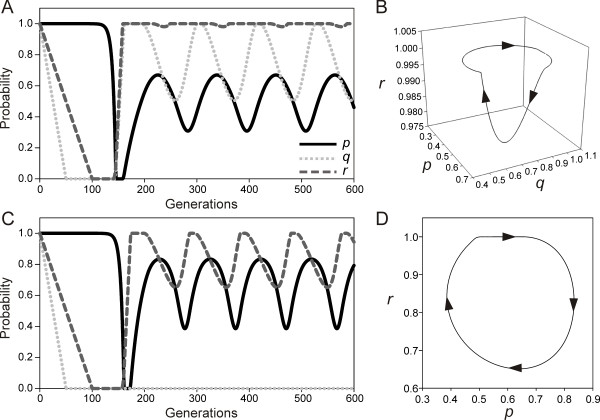
**Blackmailing leads to cyclic oscillations in the mixed strategies in the presence (A and B) and absence (C and D) of mate guarding**. (A) In the presence of mate guarding, all three traits, i.e. monogamy, mate guarding and policing show stable oscillations which form limit cycle (B) in *p*-*q*-*r *space. (C) In the absence of mate guarding monogamy and policing still oscillate in stable oscillations, which form a limit cycle (D) in *p*-*r *space. All parameter values are as per Figure 2 except in (C) and (D) *y *= 0.2, *s *= 0. Arrows indicate direction of change.

If both punishment and blackmailing options are present (*y *> 0, *a *> 0) then no single strategy is an ESS, given that the punishment is greater than the advantage of polygamy, i.e. *y *>*z*(*1-β-s*), and the benefit of blackmailing is more than the cost of policing, *a *>*c_P _*(Figure 2C, 3A). In this case the probabilities *p*, *q *and *r *keep on oscillating. We can analyze this from equations (3), (4) and (5). When *p *is small, derivative of *r *is positive as policing individuals get side payments in terms of blackmailing. However, as *p *increase, both *q *and *r *decrease as there is no cheater to guard or police so the cost of guarding and policing was worthless. However, at low levels of mate guarding and policing, polygamy increases which further encourages guarding and policing traits in the population and the cycle continues (Figure 3B).

In the above three situations we considered functional mate guarding strategy that conferred a non zero positive benefit *s *to the guarding individual. We will now consider that there is no guarding (*s *= 0), however, there are punishment and blackmailing options (*y *> 0, *a *> 0). If the punishment is sufficiently large, i.e. *y *>*z*(*1-β*), and the benefit of blackmailing is more than the cost of policing, *a *>*c_P _*both monogamy and policing traits oscillate and monogamy is maintained at high frequency (Figure 3C). The result can be studied analytically by considering equations (3) and (5). When *r *is large and *y *>*z*(*1-β*), *p *evolves and goes to unity. When *p *is unity, derivative of *r *is negative as *c_P _*> 0. Once *r *decreases *p *also decreases. Decrease in *p *again increases *r *and the cycle continues (Figure 3D). This indicates that punishment and policing-blackmailing are both necessary and sufficient for the oscillatory coexistence of monogamy and polygamy. Mate guarding, on the other hand is helpful by reducing the critical *y *necessary for the result but does not stabilize monogamy by itself.

In all the situations there exists an unstable equilibrium at (1,0,0). If *p *is slightly perturbed the system does not return to (1,0,0). The unstable equilibrium critically depends on the assumption that EPM is impossible without other adulterous individuals in the population. If this assumption is slightly relaxed, the unstable equilibrium vanishes.

The oscillatory behavior between strategies can be interpreted in two ways. We can consider that the proportions of strategies used by mixed strategy players oscillate, however, since *p*, *q*, *r *are population frequencies, at the oscillatory equilibrium we can also consider that different pure strategies coexist where the total proportion of monogamous individuals, guarding individuals and policing individuals is *p*, *q *and *r *respectively.

### Constraints on blackmailing

**Figure 4:**
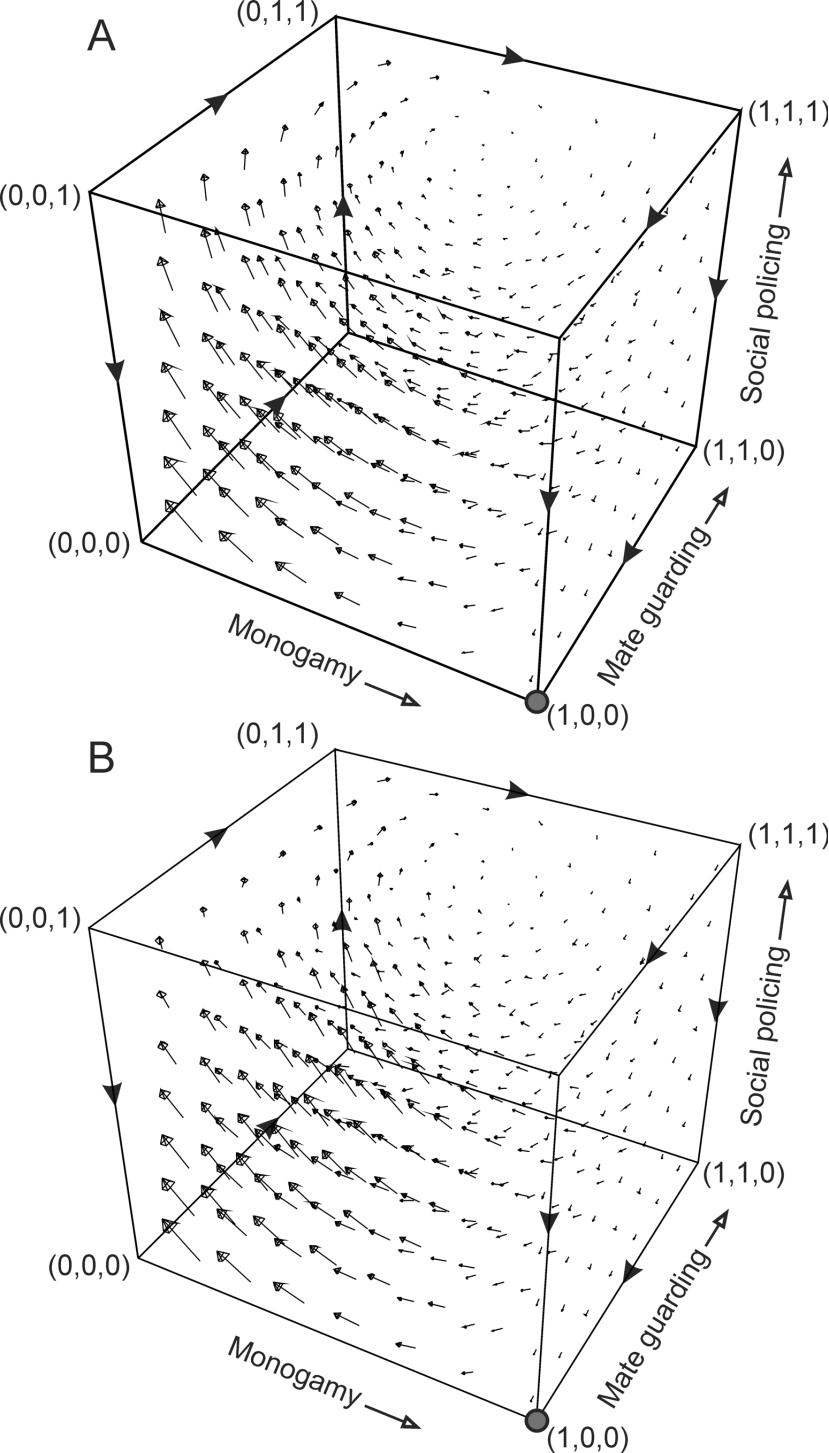
**Variations of the model to incorporate constraints on blackmailing opportunity, where (a) blackmailing opportunity decreases with increase in the mate guarding, and (b) blackmailing opportunity decreases with increase in policing individuals**. In both the cases, the results are qualitatively similar in that blackmailing leads to cyclic oscillations in strategies with no ESS similar to Figure 2C. Arrows indicate the direction of change in strategy. All parameter values as per Figure 2.

We will now refine our model to capture two scenarios (i) blackmailing opportunities decrease with increase in mate guarding and (ii) blackmailing opportunities for an individual decrease with increase in blackmailing individuals. In the basic model we considered that the opportunity to blackmail depend only upon the frequency of polygamous individuals (1-*p*), however, it can also be argued that blackmailing will decrease with mate guarding (*q*) as this would lead to decrease in EPM. We will consider that opportunity and benefits of blackmailing decrease with mate guarding by replacing '*a*' with '*a*(1-*q*)' in equation (5). Vector field plot of modified equations (Figure 4A) is qualitatively similar to Figure 2C. Furthermore, in a refined model we can also consider that the benefit of blackmailing decreases with increase in the frequency of blackmailing individuals. To capture this situation we can replaced '*a*' with '*a*(1-*r*)' in equation (5). Vector field plot of modified equation (Figure 4B) is also qualitatively similar to Figure 2C. If we consider both the refinements together by replacing *a *with *a*(1-*q*)(1-*r*) the results are still qualitatively similar to Figure 2C (results not shown). This demonstrates the robustness of the model.

### Gender differences

Assuming different costs and benefits of EPM for the two genders makes the model more complex and therefore we use a simulation approach. Simulations of the two-gender model were identical to the non-gender model if parameters for both the genders were kept identical. Introducing gender differences in the model resulted into different proportions of traits in the two genders; nevertheless, polymorphism existed in both the genders. Interestingly satisfying the condition *y *>*z *(*1-β -s*) in only one of the genders was sufficient for polymorphism in both genders. Difference in *l, z *or *y *alone or in combination led to substantial gender difference. If males were assumed to have greater losses due to partner's EPM (*l*) and additional payoff due to their own EPM (*z*) and/or smaller penalty of punishment (*y*), there was greater proportion of polygamy in males as compared to females. A difference in benefit of blackmailing (*a*), on the other hand was unable to induce substantial difference in the proportion of traits across genders. The model therefore expects that there can be gender differences in the tendency for engaging in EPM but there need not be a gender difference in the involvement in gossiping and blackmailing.

## Discussion

In humans, marriage has evolved as an institution in which interpersonal relationships are acknowledged by the society. Social pressure to reinforce faithful monogamy is predominantly and perhaps uniquely human. Active mate guarding is exhibited by many species showing bi-parental care but the element of social policing has not been demonstrated in non-humans so far. From historical times and across widely different cultures, individuals involved in EPM have been threatened by punishment ranging from community boycott to death, given that they are caught. The societal component of reinforcing monogamy is certainly language dependent. Language can help in gaining information about one's mate in one's absence given that there is social policing against EPM. Social policing is a group beneficial trait and it is less costly than mate guarding, as it is incidental to the regular activities. However, one can always free ride on the benefits gained from social policing by others without contributing to social policing. Therefore the evolution and stability of social policing is a possible paradox. The possibility of side payments from blackmailing effectively resolves the paradox. Blackmailing need not actually happen with very high frequency but the incentive to blackmail can stabilize policing and in turn monogamy in the population.

We showed that only two conditions were essential for the continued existence of obligate monogamy. First was that the maximum probabilistic cost of punishment was greater than the advantage of polygamy i.e. *y *>*z*(1-*β*-*s*) and second, the maximum probabilistic benefit of blackmailing was greater than the cost of policing (*a *>*c_P_*). We assumed that the benefits of mate guarding were more than the cost (*l.s *>*c_G_*) to stabilize mate guarding in the system. However, mate guarding alone was neither necessary nor sufficient to stabilize monogamy. Punishment and policing, stabilized by blackmailing opportunity was on the other hand both necessary and sufficient for the continued existence of obligate monogamy (Figure 2C).

At a more subtle level it may be argued that only the observation component of social policing gives blackmailing opportunities, not the gossiping component. The gossiping component would actually eliminate secrecy and make blackmailing impossible. Therefore individuals may invest only in the observational component and there would be competition for obtaining blackmailing worthy secrets but no motivation for gossiping. There would indeed be competition to discover blackmail worthy secrets, but such can be obtained in a very limited set of conditions. Whenever situation does not permit blackmailing, the information can be freely gossiped around since there is nothing to lose. This apart from accruing the broadly socially accepted good also serves the purpose of demonstrating the cost of social punishments. Social punishment relies on making the information public by gossiping. This would work as a threat useful in blackmailing. Individuals may build reputation as highly vocal and influential gossipers and such individuals pose a greater threat of initiating social punishment. Unless there is some demonstrated threat of punishment, the rare opportunity of blackmailing is unlikely to be fruitful. Gossiping therefore helps blackmailing rather than weakening it.

As per the suggestion of Houston and McNamara [43,44], where the loss and gains from EPM tally, we maintained consistency in the payoff structure for all the simulations presented in the paper. However, in reality, strict consistency may not be maintained especially when population is open with virgin individuals, immigrants, emigrants and skewed sex ratios. Our analysis suggests that whether the payoffs are consistent or non-consistent does not change the dynamics of the model so long as the conditions mentioned in the invasion dynamics and Figure 1 are obeyed.

In important issue regarding the policing and gossiping to be effective is to understand why polygamous individuals should be involved in this activity. For a polygamous individual, there is no primary incentive since the possible benefits of indirect mate guarding can be obliterated by the probability of its own exposure. However, for monogamous individuals there can be only benefit and no loss due to exposure. Therefore measures to increase the probability of exposure can be initiated by obligately monogamous individuals. However, if this happens, very soon gossiping could become an identifier of monogamy. As a result polygamous individuals will be compelled to support the gossip to pretend to be monogamous.

It can also be argued that policing individuals can be given a benefit through some collectively agreed policy that someone who discovers an EPM incident gets rewarded. However, such reward policies seldom evolve cooperation [48]. One of the basic problems is that there is a cost in giving reward. Individuals may shirk from contributing to reward and this will lead to second order free rider problem. Therefore policing cannot be stabilized by any other forms of reward.

Blackmailing is illegal and is considered as a crime liable for judiciary punishment in societies with formal judiciary system. However, prior to such central judiciary system, blackmailing might have been used as a private justice [49,50]. We therefore argue that during the evolution of mating systems, which is considered as a main driving force for the evolution of human social system [51], monogamy was maintained by social policing with opportunistic blackmailing. It can be speculated that after the advent of a formal judiciary system, the private justice of blackmailing could have been perceived as a threat to the formal judiciary system and therefore considered bad and illegal. However, formal police and judiciary systems could never replace social policing through gossiping and opportunistic blackmailing both of which are prevalent in modern societies too. Blackmailing by itself is a defection on the social policing and punishment system. But the defection itself stabilizes cooperation, a result similar to that of Dahanukar and Watve [52] who showed that in classical PD, refinement of defection strategies stabilizes cooperation.

In our baseline model we assumed that there is no gender difference in benefits of EPM and losses due to partner's EPM. The introduction of gender differences in the model results into different proportions of strategies in the two genders at a given time but stable or oscillating polymorphism is seen in both the genders provided our key conditions, *y *>*z*(1-*β*-*s*) and *a *>*c_P_*, are obeyed by at least one gender. This is interesting since in many societies there is a gender bias in punishment for cuckoldry. The model shows that punishment can be effective in spite of the gender bias, although it is not a justification for having a gender bias. It is known that the psychology of deception, the nature of gains and losses from EPM and the nature of sexual jealousy vary between sexes [20,21] and the model allows for these differences.

Our model is on the interface of genetic and cultural evolution. While the payoffs of reproduction are genetic, policing and punishment may be influenced by both genes and culture and further superimposed by the evolution of language in humans. Although the model is Darwinian, it does not predict gender stereotypes. Instead it predicts both within and between gender variability. Since some of the parameters such as the severity of punishment are decided by culture, and these parameters decide the equilibrium proportions, the model also allows for cross cultural variability. It is difficult to determine whether the observed variability in behavior with respect to EPM is intrinsic (differences in individual inclinations) or extrinsic (differences in opportunities to indulge in EPM) or both. Nevertheless the model is perhaps the first of Darwinian models that predicts intrinsic variation in inclinations towards EPM within as well as between genders.

Our model suggests that obligate monogamy can exist in the human population, which needs to be differentiated from monogamy imposed by the lack of opportunity. An obligately monogamous individual will not indulge in EPM even if opportunity permits. The model indicates that obligate monogamy is made possible by blackmailing. In reality how much is the relative contribution of obligate monogamy and that of opportunity limitation to human monogamy is not yet clear. Although monogamous breeding with biparental care exists in animal species, extra pair paternity has been demonstrated in various proportions in many species [53]. The question whether the observed monogamy in these species is obligate or opportunity limited has not yet been addressed. However, since blackmailing is uniquely human, we expect obligate monogamy in human rather than animal mating systems.

## Conclusions

The model shows that in spite of the potential advantages of EPM, intrinsically driven obligate monogamy can be maintained in a population. Mate guarding is neither necessary nor sufficient to stabilize obligate monogamy, but social policing strengthened by opportunistic blackmailing is both necessary and sufficient to stabilize obligate monogamy. Blackmailing makes policing a non-altruistic act and thereby stabilizes policing. Stability of policing is crucial for long term coexistence of multiple strategies, with faithful monogamy comprising substantial part of the population. In the absence of blackmailing polygamy with mate guarding is the only stable strategy. Although biparental care, EPM, mate guarding and punishment are shared by many species, gossiping and blackmailing make the human mating system unique.

## Authors' contributions

MW and AD developed the model. BG and AK contributed to conceptual development in the early stages of model. MW, AD and ND analyzed the model and performed simulations. MW and ND wrote the paper. All authors read and approved the manuscript.
